# Correlation between Dopamine Transporter Degradation and Striatocortical Network Alteration in Parkinson’s Disease

**DOI:** 10.3389/fneur.2017.00323

**Published:** 2017-07-17

**Authors:** Wei-Che Lin, Hsiu-Ling Chen, Tun-Wei Hsu, Chien-Chin Hsu, Yung-Cheng Huang, Nai-Wen Tsai, Cheng-Hsien Lu

**Affiliations:** ^1^Department of Diagnostic Radiology, Kaohsiung Chang Gung Memorial Hospital, Chang Gung University College of Medicine, Kaohsiung, Taiwan; ^2^Department of Radiology, Taipei Veterans General Hospital, Taipei, Taiwan; ^3^Department of Nuclear Medicine, Kaohsiung Chang Gung Memorial Hospital, Chang Gung University College of Medicine, Kaohsiung, Taiwan; ^4^Department of Neurology, Kaohsiung Chang Gung Memorial Hospital, Chang Gung University College of Medicine, Kaohsiung, Taiwan

**Keywords:** dopamine transporter imaging, functional connectivity, movement disorder, magnetic resonance imaging, parkinsonism, ^99m^Tc-TRODAT-1 SPECT/CT

## Abstract

The association between dopamine neuron loss and functional change in the striatocortical network was analyzed in 31 patients with Parkinson’s disease (PD) [mean disease duration 4.03 ± 4.20 years; Hoehn and Yahr (HY) stage 2.2 ± 1.2] and 37 age-matched normal control subjects. We performed ^99m^Tc-TRODAT-1 SPECT/CT imaging to detect neuron losses and resting-state functional magnetic resonance imaging to detect functional changes. Mean striatal dopamine transporter binding ratios were determined by region of interest analysis. The functional connectivity correlation coefficient (fc-cc) was determined in six striatal subregions, and interactions between these binding ratios and the striatocortical fc-cc values were analyzed. The PD patients had significant functional network alterations in all striatal subregions. Lower striatal dopamine transporter binding correlated significantly with lower fc-cc values in the superior medial frontal (SMF) lobe and superior frontal lobe and higher fc-cc values in the cerebellum and parahippocampus. The difference in fc-cc between the ventral inferior striatum and SMF lobe was significantly correlated with increased disease duration (*r* = –0.533, *P* = 0.004), higher HY stage (*r* = –0.431, *P* = 0.020), and lower activities of daily living score (*r* = 0.369, *P* = 0.049). The correlation of frontostriatal network changes with clinical manifestations suggests that fc-cc may serve as a surrogate marker of disease progression.

## Introduction

Parkinson’s disease (PD) is a common neurodegenerative disease associated with dopamine-related movement disorders. The substantia nigra and basal ganglia, both innervated by dopaminergic neurons, are seriously affected in PD (1), and are commonly assessed to determine disease diagnosis, disease severity, and the best treatment (2). ^99m^Tc-[2-[[2-[[[3-(4-chlorophenyl)-8-methyl-8-azabicyclo [3,2,1] oct-2-yl] methyl] (2-mercaptoethyl) amino] ethyl] amino]-ethanethiolato(3-)-N2,N2,S2, S2]oxo-[1R-(exo-exo)] (^99m^Tc-TRODAT-1) is a specific tracer developed to bind selectively to dopamine transporters in the brain. Uptake ratio of ^99m^Tc-TRODAT-1 in the striatum as revealed by single-photon emission computed tomography (SPECT) allows for *in vivo* assessment of pre-synaptic dopaminergic neuron activity. Decreased striatal tracer uptake, indicating the loss of dopaminergic neurons, has been used to evaluate disease progression and confirm the presence of symptomatic lesions in the early stage of PD (3).

In addition to its involvement in movement control, the striatum is also the gatekeeper for the reciprocal flow of information between neocortical and subcortical structures (4), and functionally distinct neural circuits have been found to be associated with specific striatal subdivisions based on their cortical region distributions (5). In addition to manifesting characteristic motor impairment, patients with PD also have a wide range of non-motor symptoms that reflect dysfunction beyond the nigrostriatal pathway (6). These symptoms impact sleep, mood, and cognition, and include fatigue, pain, and autonomic disorders that adversely affect quality of life (7). It is believed that dopamine neuron loss in the striatum contributes to the dysregulation or disruption of functional interactions between the striatum and other neocortical areas (8–10). However, it is impossible to evaluate functional network changes as a consequence of histologically verifiable dopamine neuron loss directly. A better understanding of the pathophysiology of PD is essential for its treatment and prevention.

Using resting-state functional magnetic resonance imaging (rs-fMRI) to determine deviations from normal coherent spontaneous blood oxygen level-dependent activity offers a means of assessing the status of functional systems within the brain without the interpretive confound of variable task performance (11). Using rs-fMRI, Di Martino et al. demonstrated distinct functional connectivity profiles of striatal subdivisions that correspond to the hypothesized motor, cognitive, and affective divisions in the human striatal system (12). It was also found that dopamine neuron loss leads to a remapping of cerebral connectivity due to the reduction in spatial segregation between different corticostriatal loops (8). It is thus reasonable to hypothesize that dopamine neuron depletion in PD can change the particular functional networks needed for motor and non-motor integration.

To test this hypothesis, we used ^99m^Tc-TRODAT-1 to evaluate the striatal dopamine neuron density in PD and rs-fMRI to delineate shared and distinct functional networks in six striatal subdivisions [the bilateral dorsal caudate (DC), superior ventral striatum, inferior ventral striatum, dorsal caudal putamen (DCP), dorsal rostral putamen (DRP), and ventral rostral putamen (VRP)] (12) and other cortical areas, and to evaluate their relationships as striatal dopamine terminal function declines. Finally, possible relationships between those dopamine-related striatocortical networks and clinical variables were also examined. The goal of this study was to determine how and where dopamine neuron loss impacts the pattern of segregated functional networks in striatal subdivisions in PD.

## Materials and Methods

### Participants

The study was approved by the Local Ethics Committee on Human Research of Kaohsiung Chang Gung Memorial Hospital in Taiwan. All the participants or their next of kin provided written informed consent prior to participation in the study. Thirty-one right-handed PD patients (17 men and 14 women, mean age: 60.39 ± 13.03 years) with no previous history of neurological or psychiatric illnesses, psychotropic medication, or contraindication to magnetic resonance imaging (MRI) were prospectively enrolled at the Neurology Department of Kaohsiung Chang Gung Memorial Hospital. Patients were included if they had idiopathic PD diagnosed by an experienced neurologist using the Parkinson’s Disease Society’s criteria for idiopathic PD (13). Of the 31 PD patients, 12 had never used any PD medication and 19 were taking dopaminergic medications (e.g., levodopa and dopamine agonists). The disease severity and functional status of each patient were evaluated on the Unified Parkinson’s Disease Rating Scale (UPDRS) (14), the modified Hoehn and Yahr scale (HY stage) (15), and the Schwab and England activities of daily living (SE-ADL) scale (16) in the “OFF” state, which refers to the period of lower levodopa effectiveness in eliminating motor symptoms and consequent inability to function properly.

The mean disease duration, defined as the time since symptoms first became subjectively apparent, was 4.03 ± 4.20 years. For comparison, 37 sex- and age-matched healthy subjects [24 men and 13 women, mean age: 55.49 ± 8.05 years; normal control (NC) group] with no medical history of neurologic diseases or psychiatric illnesses, alcohol/substance abuse, or head injury, and with similar levels of education, were also recruited.

### Neuropsychological Tests

The Mini-Mental State Examination (MMSE) (17) and Cognitive Ability Screening Instrument (CASI) (18) were administered to all the subjects. The CASI has a score range of 0–100 and provides a quantitative assessment of attention, concentration, orientation, short-term memory, long-term memory, language abilities, visual construction, list-generating fluency, abstraction, and judgment. The typical administration time for the CASI is 15–20 min.

### Tc-99m TRODAT-1 Brain SPECT/CT Data Acquisition and Preprocessing

#### Data Acquisition, Image Processing, and Region of Interest (ROI) Delineation

The examination of Tc-99m TRODAT-1 Brain SPECT/CT imaging, post-processing, and ROI evaluation in patients with PD were conducted as previously reported (19) and are summarized in Supplementary Material.

Each patient with PD was intravenously injected with a 925-MBq (25 mCi) dose of Tc-99m TRODAT-1 (Institute of Nuclear Energy Research, Lung-Tan, Taiwan). The brain SPECT and CT scans were done 4 h later consecutively, using a hybrid SPECT/CT system (Symbia T; Siemens Medical Solutions, Hoffman Estates, IL, USA) with the given patient lying stably in a supine position with the head resting in a holder.

The CT images were reconstructed for SPECT attenuation correction, image fusion, and ROI delineation. Reorientation with sagittal slices parallel to the anterior commissure–posterior commissure line and correction for transaxial and coronal slice deviation was done manually by inspecting the SPECT images. Three consecutive SPECT transaxial slices showing the highest striatal uptake were summed. For manual SPECT ROI delineation, ROIs of the striatum, caudate, putamen, and occipital cortex were manually delineated directly on the summed SPECT image. The principal investigator reviewed all of the SPECT scans to determine the ROI delineation. In equivocal cases, a second observer also conducted a review. Both were blinded to the laboratory results at the time of clinical and imaging assessment.

#### Striatal and Subregional TRODAT-1 BP_ND_

We used the low DAT concentration area of the occipital cortex as a background ROI. The striatal, caudate, and putamen non-displaceable binding potential (BP_ND_) values were calculated by subtracting the mean counts of the occipital cortex ROI from the mean counts of the striatal, caudate, and putamen ROIs and dividing the result by the mean counts of the occipital cortex ROI (19).

### MR Data Acquisition and Preprocessing

#### Data Acquisition

MRI scanning was performed on a 3.0-T GE Signa MRI whole body scanner (GE Medical Systems, Milwaukee, WI, USA). Functional resting-state images from 300 contiguous echo planar imaging whole brain functional scans (TR: 2 s, TE: 30 ms, FOV: 240 mm, flip angle 80°, matrix size 64 × 64, thickness 4 mm) were collected. During the resting experiment, the scanner room was darkened and the participants were instructed to relax, with their eyes closed, without falling asleep. To coregister and normalize functional images with a standardized template, a 3D high-resolution T_1_-weighted anatomical image was also acquired using an inversion recovery fast spoiled gradient-recalled echo pulse sequence (TR: 9.5 ms; TE: 3.9 ms; TI: 450 ms; flip angle 20°; field of view 256 mm; matrix size 512 × 512).

#### Resting-State Functional MRI Preprocessing and Individual Analyses

Resting-state fMRI data preprocessing was then performed using the Statistical Parametric Mapping software package (SPM8, Wellcome Department of Cognitive Neurology, London, UK; http://www.fil.ion.ucl.ac.uk/spm/) and Data Processing Assistant for Resting-State fMRI tools (20). All of the image processing procedures were conducted according to the procedures detailed in a previous study (21) and are summarized in Supplementary Material.

### Functional Connectivity Analysis

#### ROI Selection and Seed Generation

We generated a spherical seed ROI (diameter = 5 mm), as described by Di Martino et al. (12), in each of six areas of the striatum including the ventral caudate (superior, VSs), ventral caudate/nucleus accumbens (inferior, VSi), DC, DCP, DRP, and VRP, respectively, in each of the two hemispheres, with MNI coordinates centered at *x* = (±) 9, *y* = 9, *z* = −8; *x* = (±) 10, *y* = 15, *z* = 0; *x* = (±) 13, *y* = 15, *z* = 9; *x* = (±) 28, *y* = 1, *z* = 3; *x* = (±) 25, *y* = 8, *z* = 6; and *x* = (±) 20, *y* = 12, *z* = −3, respectively. All the seed ROIs were centered on gray matter foci (using the 152 brain standard MNI gray matter template provided by FSL).

#### Functional Connectivity Map

For each participant, the seed (resting-state time series) of each ROI was obtained by averaging the time series of all the voxels within the ROI, and its correlation with the remaining whole brain was analyzed in a voxel-wise manner. The correlation coefficients were then transformed to *z* values using the Fisher *r*-to-*z* transformation to improve normality. The voxel values of spatial maps represented the strength of the correlations with the ROIs.

#### Group-Level Analysis

The individual *z* values were entered into one-sample *t* tests in a voxel-wise manner using the SPM8 package to determine the brain regions that had significant positive connectivity with each ROI within each group. The significance threshold was set at *P*_corrected_ < 0.05 based on family-wise error. For each seed ROI, thresholded functional connectivity values from both groups were combined by means of an "OR" operation, and then used as masks in between-group two-sample *t* tests.

To compare the functional connectivity of each striatal seed between groups, the *z*-values were entered into a two-sample *t*-test in a voxel-wise manner. This analysis was masked using the combined positive functional connectivity maps for each striatal seed, as discussed above. Statistical significance was determined with a voxel-wise *P*_corrected_ < 0.05 based on Monte Carlo simulations (22). The corrected threshold corresponded to *P*_uncorrected_ < 0.01, with a minimum cluster size of 40 voxels for multiple comparisons. The mean strength of the functional connectivity of correlation coefficient (fc-cc) from the clusters with a significant group main effect was extracted for further correlation analysis.

### Statistical Analysis

The demographic data, including age, sex, and education, were compared among the study groups using a two-sample Student’s *t*-test and Pearson chi-square test, where appropriate, and are reported as the mean ± the SD. Differences in neuropsychological assessments were analyzed using an analysis of covariance model with the participant’s age, sex, and education values as covariates.

#### Correlations between Striatal TRODAT-1 BP_ND_ Values and Functional Connectivity Map Differences in PD Group (Dopamine-Associated Striatal Functional Networks)

To investigate the association between the striatal SPECT uptake values and the two-sample connectivity map difference for each striatal seed in the PD patients, the correlation of the fc-cc value of significant clusters in two-sample results with the striatal TRODAT-1 BP_ND_ value was evaluated using Pearson correlation. The threshold for statistical significance was set at *P* < 0.05 with Bonferroni correction for multiple comparisons.

#### Correlation between Dopamine-Associated Striatal Functional Networks and Clinical Assessments

The correlation of dopamine-related striatal functional networks with disease duration, disease severity, and neuropsychological test results was assessed using partial Pearson correlation with the participant’s age, sex, and education values as covariates. The threshold for statistical significance was set at *P* < 0.05 with Bonferroni correction for multiple comparisons. All statistical analyses of demographic data, neuropsychological assessments, and global tissue volumes were performed using SPSS software, version 17, for Windows (SPSS, Chicago, IL, USA).

## Results

### Demographic and Clinical Characteristics

The demographic and clinical data of the participants are shown in Table 1. Sex, age, and education level were similar between the PD patients and the NC group. The PD patients had a mean total UPDRS score of 41.6, a mean modified HY stage of 2.2, and a mean SE-ADL score of 80.7.

**Table 1:** Demographic variables, clinical variables, and single-photon emission computed tomography uptake measurements compared between patients with PD and NCs.

Variable	PD group (*n* = 31)	NC group (*n* = 37)	*P* value
Age (years)	60.39 ± 13.03	55.49 ± 8.05	0.175^a^
Sex (male/female)	17/14	24/13	0.461^b^
Education (years)	9.13 ± 5.20	10.97 ± 5.15	0.138^a^
MMSE	22.43 ± 6.20	27.59 ± 2.35	<0.001*^,c^
CASI	77.96 ± 21.31	89.70 ± 8.19	0.002*^,c^
Disease duration (years)	4.03 ± 4.20		
UPDRS I	3.87 ± 3.40	–	–
UPDRS II	12.10 ± 8.95	–	–
UPDRS III	25.68 ± 16.44	–	–
UPDRS total score	41.55 ± 27.62	–	–
Modified HY stage^d^	2.18 ± 1.22	–	–
SE-ADL^e^	80.65 ± 18.96	–	–
**Striatal and subregional TRODAT-1 BP_ND_**			
Right caudate	1.57 ± 0.20		
Right putamen	1.47 ± 0.22		
Right striatum	2.02 ± 0.20		
Left caudate	1.90 ± 0.18		
Left putamen	2.09 ± 0.20		
Left striatum	2.01 ± 0.17		

*Means and SDs of raw scores for the patients with PD and the NC groups. For each variable, the P value indicates the significance level of the appropriate statistical test comparing the raw scores of the NC group and the patients with PD*.

*MMSE, Mini-Mental State Examination; modified HY stage, modified Hoehn and Yahr stage; PD, Parkinson’s disease; SE-ADL, Schwab and England activities of daily living scale; CASI, Cognitive Ability Screening Instrument; UPDRS, Unified Parkinson’s Disease Rating Scale*.

*^a^Two-sample unpaired t-test*.

*^b^Chi-square test*.

*^c^Analysis of covariance test which adjusted for age, sex, and education*.

*^d^For modified HY stage, the maximum stage is 5*.

*^e^For the SE-ADL, the minimum score is 0, suggesting vegetative functions; the maximum score is 100, suggesting complete independence*.

*P value < 0.05.

### Neuropsychological Tests between Groups

The PD group revealed worse cognition, including lower MMSE (22.43 ± 6.20 versus, 27.59 ± 2.35; *P* < 0.001) and CASI scores (77.96 ± 21.31 versus 89.70 ± 8.19; *P* = 0.002) than the NC group (Table 1).

### Striatal and Subregional Tc-99m TRODAT-1 BP_ND_ in PD Group

The striatal and subregional TRODAT-1 BP_ND_ results are presented in Table 1. The average TRODAT-1 BP_ND_ value was 1.57 ± 0.20 in the right caudate, 1.47 ± 0.22 in the right putamen, 2.02 ± 0.20 in the right striatum, 1.90 ± 0.18 in the left caudate, 2.09 ± 0.20 in the left putamen, and 2.01 ± 0.17 in the left striatum.

### Differences in Subregional Striatal Functional Connectivity

**Figure 1:**
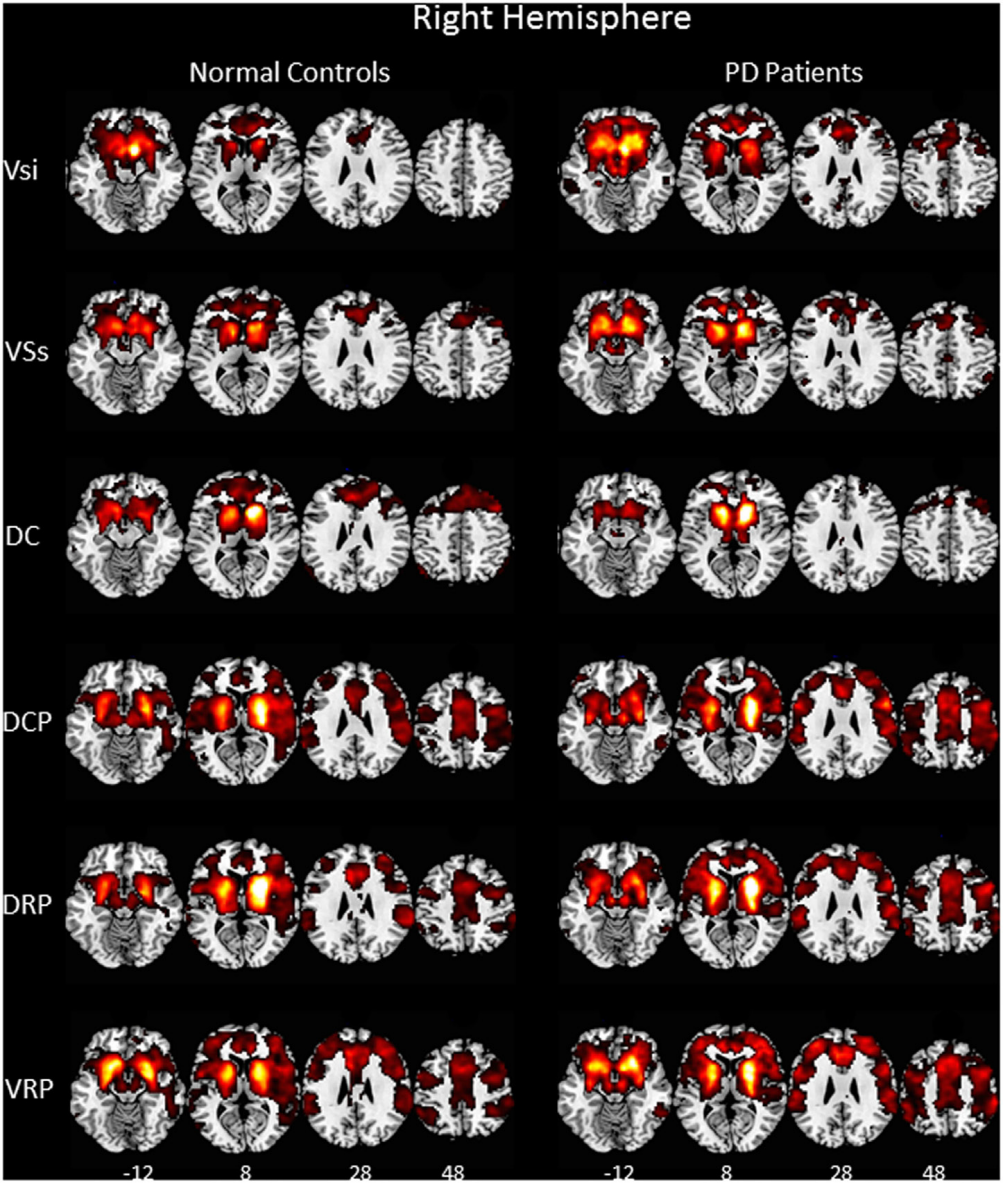
Functional connectivity of six right hemisphere striatal seeds. Pattern of significantly positive relationships for VSs, VSi, DC, DCP, DRP, and VRP in which the MNI coordinates were centered at *x* = 9, *y* = 9, *z* = −8; *x* = 10, *y* = 15, *z* = 0; *x* = 13, *y* = 15, *z* = 9; *x* = 28, *y* = 1, *z* = 3; *x* = 25, *y* = 8, *z* = 6; and *x* = 20, *y* = 12, *z* = −3, respectively, from top to bottom in the normal controls and in the patient with Parkinson’s disease (*P* < 0.001, FWE corrected). The patterns of functional connectivity of the six striatal seeds were bilaterally similar. Therefore, the pattern of the right hemisphere is represented. Abbreviations: VSi, ventral caudate/nucleus accumbens (inferior); VSs, ventral caudate (superior); DC, dorsal caudate; DCP, dorsal caudal putamen; DRP, dorsal rostral putamen; VRP, ventral rostral putamen.

**Figure 2:**
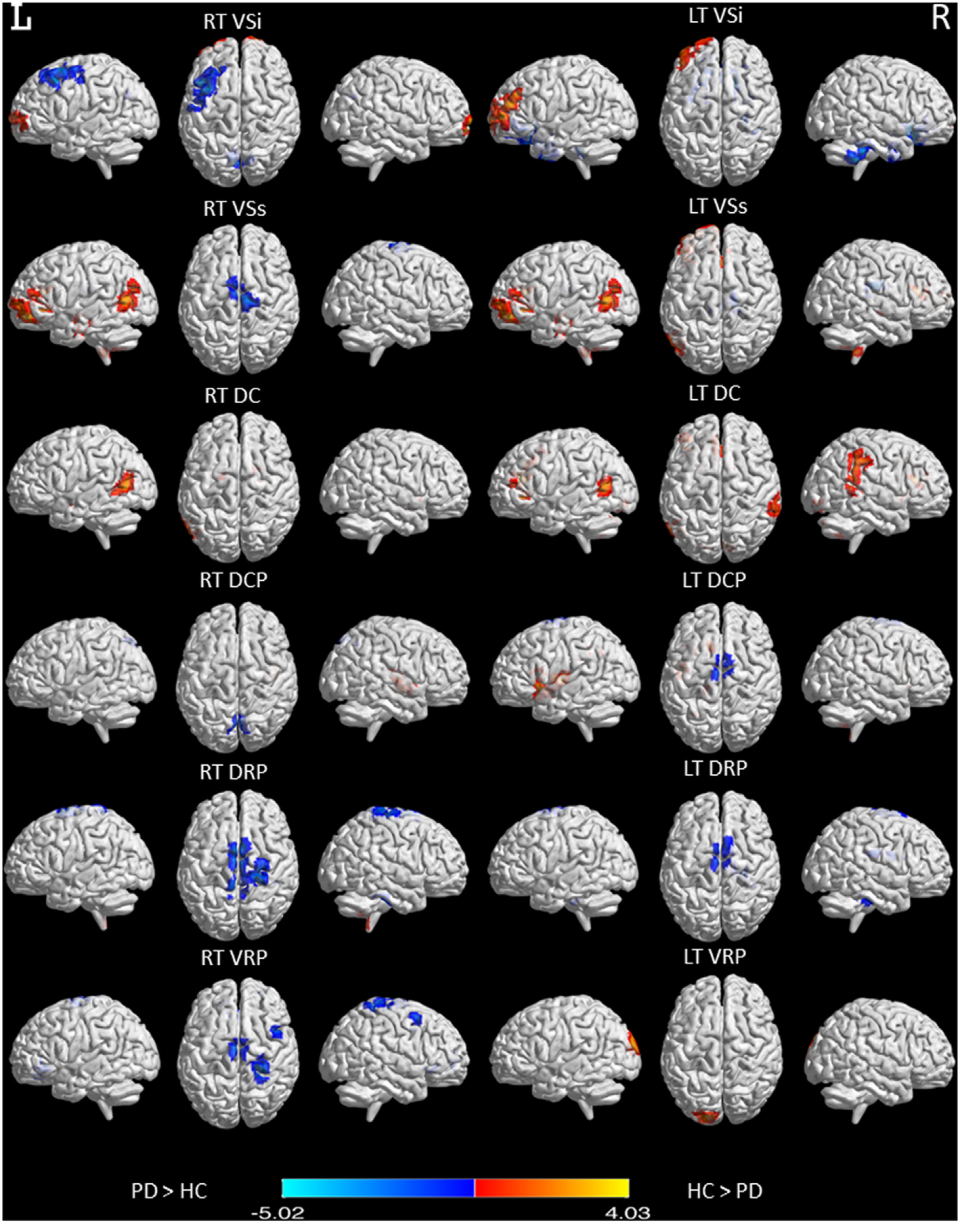
Functional connectivity differences in six striatal subregions between PD and normal control (NC) participants. Maps showing significant functional connectivity differences in six striatal subregions between the PD and NC participants are rendered on a structural T1 magnetic resonance template. Statistical significance was determined with a voxel-wise *P*_corrected_ < 0.05 based on Monte Carlo simulations. The corrected threshold corresponds to *P*_uncorrected_ < 0.01 with a minimum cluster size of 40 voxels for multiple comparisons.

Our rs-fMRI analysis demonstrated that functional connections were segregated in the striatum and roughly parallel in the neocortices of both the NC group and the PD patients (Figure 1). Two-sample *t*-testing clearly showed significant between-group differences in the functional connectivity maps of each striatal seed (Figure 2) (*P* < 0.05, AlphaSim corrected). The hot and cold colors in the functional connectivity maps projected onto the surface of the normalized brain template indicated that the NC group had some functional correlation maps that were greater than and others that were smaller than those of the PD patients. Spatial maps and anatomical descriptions of each seed can be found in Table 2.

**Table 2:** Significant differences in cluster functional connectivity between PD and normal controls (NCs).

Seed	Functional maps	Cluster anatomical locations	Cluster size	Primary peak location (MNI)			*T*-value
				*x*	*y*	*z*	
RT VSi	NC > PD	Right, SF gyrus, medial	75	12	72	0	4.265
		Left, SF gyrus	59	−24	66	3	2.840
LT Vsi		Left, middle frontal gyrus	203	−48	42	2	4.030
RT VSs		None					
LT VSs		Left, SF gyrus, medial	83	−6	54	3	3.894
		Right, cerebellar lobule IX	152	3	−57	−54	3.781
		Left, inferior frontal gyrus, orbital part	276	−39	36	−3	3.573
		Left, middle temporal gyrus	157	−42	−57	0	3.317
		Left, middle temporal gyrus	62	−51	−21	−1	3.085
		Right, anterior cingulate and paracingulate gyri	60	6	27	18	3.039
RT DC		Right, lenticular nucleus, pallidum	88	24	0	−3	3.586
		Left, middle temporal gyrus	76	−57	−66	6	3.246
		Left, lenticular nucleus, pallidum	63	−21	−9	3	3.079
LT DC		Right, lenticular nucleus, pallidum	156	18	3	3	3.729
		Left, inferior frontal gyrus, orbital part	77	−39	33	−3	3.660
		Right, cerebellar lobule IX	73	6	−60	54	3.534
		Left, anterior cingulate and paracingulate gyri	138	−6	36	21	3.552
		Left, middle temporal gyrus	135	−54	−66	12	3.346
		Right, supramarginal gyrus	80	63	−42	42	3.477
		Right, cerebellar crus II	88	3	−84	−30	3.272
RT DCP		Right, insula	44	45	0	−6	3.289
LT DCP		Left, rolandic operculum	73	−42	−6	3	3.689
		Left, angular gyrus	82	−27	−51	33	3.622
		Right, cerebellar lobule IX	71	9	−54	−57	3.470
		Left, insula	120	−36	15	−3	3.279
RT DRP		Right, cerebellar lobule IX	44	6	−51	–57	3.183
LT DRP		None					
RT VRP		None					
LT VRP		Left, superior occipital gyrus	104	−18	−96	27	4.082
RT VSi	PD > NC	Left, middle frontal gyrus	106	−48	18	48	3.479
		Right, cuneus cortex	50	12	−72	36	3.052
LT Vsi		Right, rectus gyrus	912	9	15	−24	5.018
		Right, cerebellar lobule VIII	233	39	−42	−48	4.679
		Left, parahippocampus	120	−24	6	−45	4.304
		Right, inferior temporal gyrus	81	39	0	−39	3.719
RT VSs		Left, supplementary motor area	80	0	−9	72	3.321
LT VSs		Right, thalamus	119	15	−24	18	4.184
RT DC		None					
LT DC		None					
RT DCP		Right, precuneus	40	3	−66	48	2.782
LT DCP		Left, supplementary motor area	54	−3	−12	78	3.359
RT DRP		Left, supplementary motor area	336	0	−6	72	3.765
		Right, caudate nucleus	73	9	12	−12	3.224
		Right, cerebellar lobule VI	47	45	−33	−30	2.883
LT DRP		Right, supplementary motor area	66	6	−9	78	3.265
		Right, cerebellar lobule VI	44	42	−33	−33	3.040
		Right, caudate nucleus	44	18	9	21	3.039
RT VRP		Left, anterior cingulate and paracingulate gyri	77	−6	30	–3	3.833
		Right, caudate nucleus	50	9	12	−12	3.827
		Right, postcentral gyrus	97	27	–36	72	3.336
		Right, superior frontal gyrus, orbital part	41	27	48	–3	3.645
		Right, middle frontal gyrus	42	45	12	54	3.298
LT VRP		None					

*The statistical threshold was set to *P*_corrected_ < 0.05 (AlphaSim correction), with corrected cluster significance of *P*_uncorrected_ < 0.01 corresponding to at least 40 contiguous voxels*.

*Voxel size = 3 mm × 3 mm × 3 mm*.

*VSs, ventral caudate (superior); VSi, ventral caudate/nucleus accumbens (inferior); DC, dorsal caudate; DCP, dorsal caudal putamen; DRP, dorsal rostral putamen; VRP, ventral rostral putamen; L, left; R, right; AAL, automated anatomical labeling; MNI, Montreal Neurological Institute; SF, superior frontal*.

### Correlations between Striatal TRODAT-1 BP_ND_ Values and Functional Connectivity Map Differences in PD Group (Dopamine-Related Striatal Functional Networks)

**Figure 3:**
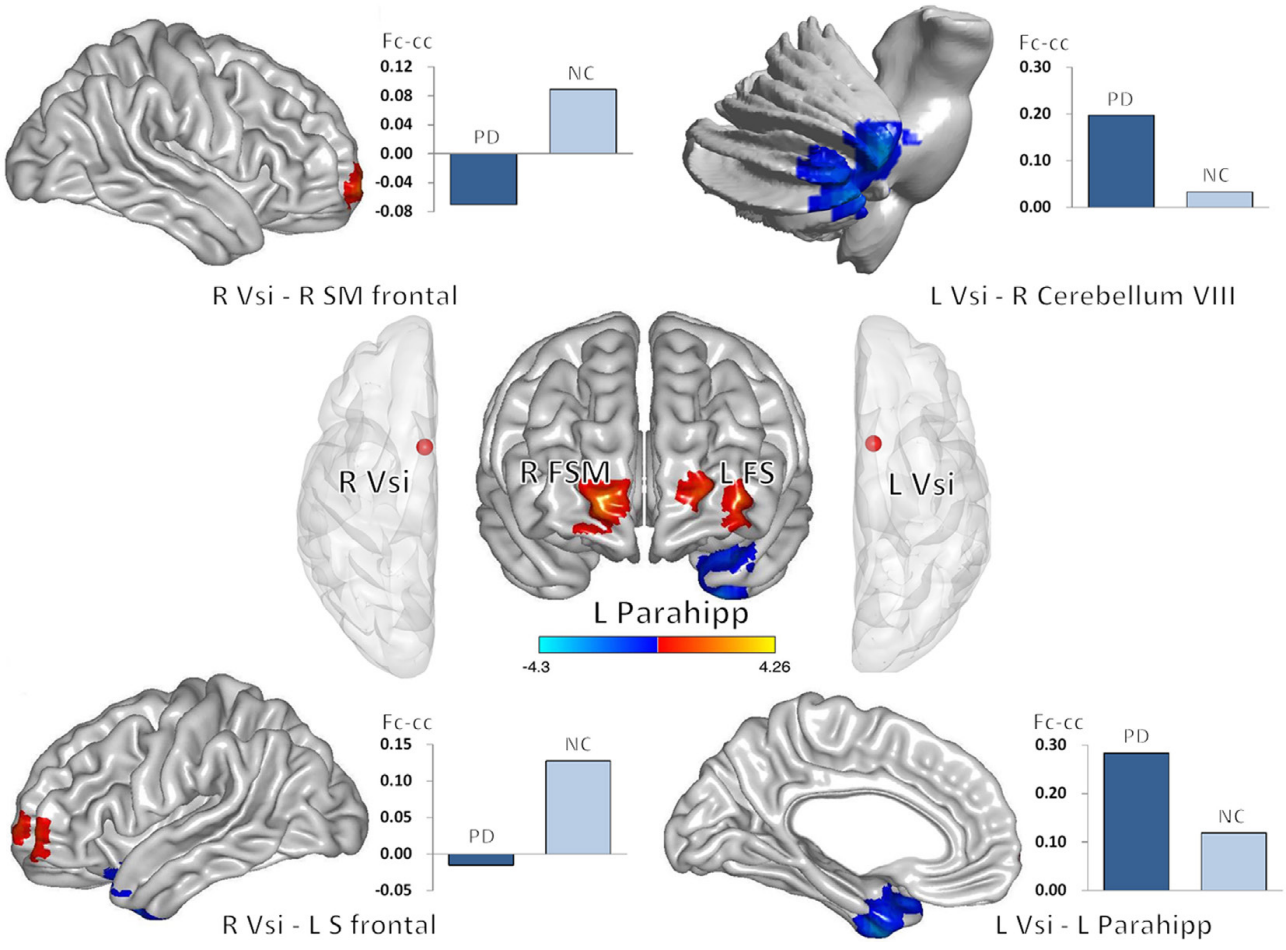
Correlations between decreased striatal TRODAT-1 BP_ND_ and striatocortical network alterations. Lower striatal TRODAT-1 BP_ND_ was correlated with lower Vsi functional connectivity with the right superior medial frontal lobe and left superior frontal lobe. Lower striatal TRODAT-1 BP_ND_ was also correlated with higher Vsi functional connectivity with the right cerebellum and left parahippocampus. The correlations were significant at the 0.05 level using a Pearson correlation analysis (two tailed). Red and blue colors indicate positive and negative correlation regions, respectively. Abbreviations: Fc-cc, functional connectivity correlation coefficient; FS, superior frontal gyrus; FSM, superior frontal gyrus medial; L, left; Parahipp, parahippocampus; R, right; Vsi, ventral caudate/nucleus accumbens (inferior).

Decreased striatal and subregion TRODAT-1 BP_ND_ values were associated with changes in fc-cc in 4 regions of the bilateral Vsi functional network (Table 3; Figure 3). The lower fc-cc between the Vsi and right superior medial frontal (SMF) lobe was correlated with lower TRODAT-1 BP_ND_ values in the right striatum, right putamen, left striatum, left caudate, and left putamen. The lower fc-cc between the Vsi and left superior frontal lobe was correlated with lower TRODAT-1 BP_ND_ values in the left striatum and left caudate.

**Table 3:** Tc-99m TRODAT-1 SPECT/CT-associated striatal functional networks.

Striatal functional networks				
Seeds	R VSi	R VSi	L VSi	L VSi
Brain regions	R SMF lobe	L SF lobe	R cerebellum	L parahippocampus

**TRODAT**				

R_striatum	0.412 (0.021)*	0.282 (0.125)	−0.221 (0.232)	−0.303 (0.097)
R_caudate	0.274 (0.136)	0.263 (0.152)	−0.310 (0.089)	−0.393 (0.029)*
R_putamen	0.443 (0.013)*	0.276 (0.133)	−0.161 (0.387)	−0.246 (0.182)
L_striatum	0.489 (0.005)*	0.393 (0.029)*	−0.334 (0.066)	−0.304 (0.096)
L_caudate	0.364 (0.044)*	0.384 (0.033)*	−0.379 (0.036)*	−0.289 (0.115)
L_putamen	**0.524 (0.002**)*	0.345 (0.057)	−0.243 (0.188)	−0.335 (0.066)

*Correlation is significant at the 0.05 level by using Pearson correlation analysis (two tailed).

*Bold type: P < 0.05, with Bonferroni correction for multiple comparisons*.

*VSi, ventral caudate/nucleus accumbens (inferior); SMF, superior medial frontal; SF, superior frontal; L, left; R, right*.

The higher fc-cc between the Vsi and right cerebellum was correlated with lower a TRODAT-1 BP_ND_ value in the left caudate. The higher fc-cc between the Vsi and left parahippocampus was correlated with a lower TRODAT-1 BP_ND_ value in the right caudate.

Among the aforementioned fc-cc values, the fc-cc values between the Vsi and right SMF lobe was correlated with lower TRODAT-1 BP_ND_ values in the bilateral striatum and bilateral putamen based on a threshold of *P* < 0.05 corrected for multiple comparisons.

### Correlation between Dopamine-Related Striatal Functional Networks and Clinical Assessment

**Figure 4:**
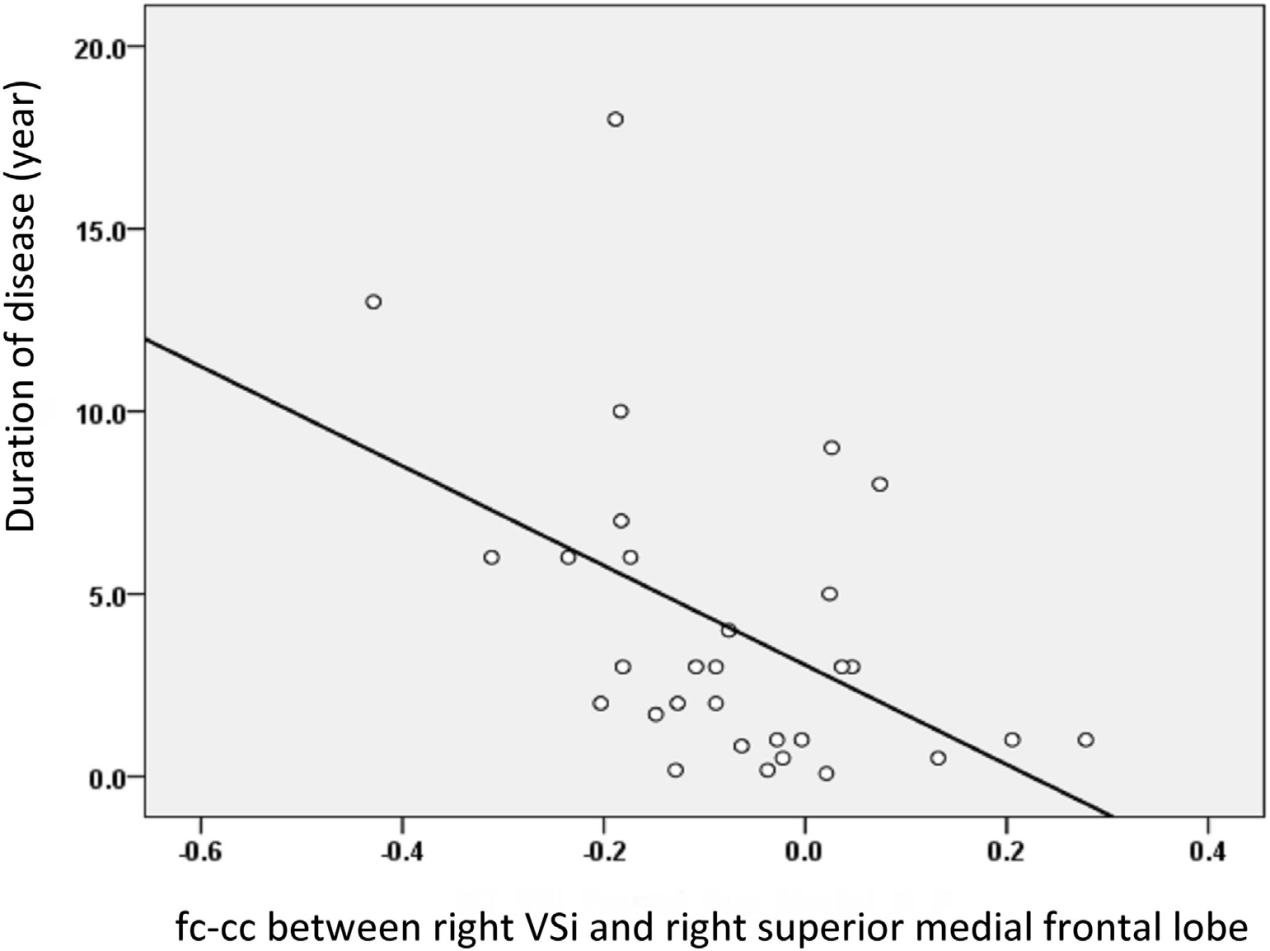
The relationship between the fc-cc of the Vsi-superior medial frontal (SMF) lobe and duration of disease. The decreased connectivity between the Vsi and SMF lobe was associated with increased disease duration. Abbreviations: fc-cc, functional connectivity correlation coefficient.

The fc-cc difference between the Vsi and SMF lobe was also significantly correlated with increased disease duration (*r* = −0.533, *P* = 0.004) (Figure 4), higher HY stage (*r* = −0.431, *P* = 0.020), and lower SE-ADL scale score (*r* = 0.369, *P* = 0.049). Using a *P* value of 0.05 corrected for multiple comparisons, the fc-cc difference between the Vsi and SMF lobe was significantly correlated with increased disease duration. There was no correlation between altered striatal functional networks and the neuropsychological test results.

## Discussion

### Summary

This resting-state functional connectivity study compared patients with idiopathic PD to age-matched control subjects. Using seed ROI manually defined bilaterally in six subdivisions of the striatum, we found: (i) lower striatal correlation with the extended frontal lobe, temporal lobe, rolandic operculum, angular gyrus, supramarginal gyrus, cingulate gyrus, striatum, insula, and cerebellum; (ii) increased striatal correlation with the cerebellum, supplementary motor cortex, parahippocampus, postcentral gyrus, thalamus, and occipital lobe; (iii) different effects on the putamen and caudate (Figure S1 in Supplementary Material), and most importantly (iv) correlation of disease severity with striatal dopamine neuron loss resulting from particular striatocortical network alterations. Our results support our previous hypothesis and provide clues to the pathophysiology of PD.

### Segregation of Striatal Functional Network

Using rs-fMRI analysis, first, we demonstrated a ventromedial-to-dorsolateral gradient in the striatum with a roughly parallel gradient in the neocortices of both the NC group and the PD patients (Figure 1) (12). The finding was consistent with our previous striatal structural covariance network findings of a correlation between the mean gray matter volume of the striatum with the gray matter volume across the entire neocortex in a voxel-wise manner (23). The high similarity in the distribution of subregional striatocortical networks between the PD and NC groups supported the hypothesis that human neural networks can be defined by synchronous neural activity, a common corticotrophic destiny, and selective vulnerability to neurodegenerative illness (24). It also supported the network degeneration hypothesis and suggested that pathological processes occurring in the striatum of PD brains affect the same striatocortical networks that co-vary in the striatum of healthy brains (24). Nevertheless, our patterns of rs-fMRI networks of the same striatal subdivisions in the NC and PD participants were wider than those mentioned in structural covariance studies conducted previously (25). The discrepancy between these results may be explained by the tendency of neuronal homeostasis to be maintained by functional plasticity instead of structural plasticity (26), unless the capacity of neurons deteriorates as a result of the disease and structural remodeling subsequently increases to compensate for functional impairment. Another explanation is that most information within striatocortical circuit models is indirectly conveyed through specific thalamic nuclei (27), which might also minimize the necessity of structural similarity between the striatum and cortex.

### Alteration of the Striatocortical Functional Network

Compared to the NC group, the PD patients showed widespread striatocortical functional network alterations (Figure 2). The caudate, especially the Vsi subregion, showed a decrease in connectivity with the frontal lobe, temporal lobe, parietal lobe, and some cerebellar regions and an increase in connectivity with the parahippocampus, some cerebellar regions, and the occipital lobe. Plausibly, the topography of the affected regions reflects the phenotype of PD, which is primarily a movement disorder with associated executive deficits (28) and visual disturbances (29). Our findings also support the association of parkinsonism with recently described circuits connecting the cerebellum to the basal ganglia (10). Functional or morphological modulations in the cerebellum are related to akinesia/rigidity, tremor, gait disturbance, dyskinesia, and some non-motor symptoms (10). We also found that underactivation of the ventral attentional network in the supramarginal gyrus, anterior cingulate cortex, and insula may underlie the characteristic impairment of attentional mechanisms in PD (30).

### Dopamine Degradation-Related Striatocortical Network Alterations

Change in the striatocortical network was demonstrated, for the first time, to be linked to decline in the dopamine transporter ratio of the striatum, suggesting that change in striatal dopamine activity profoundly influences corticostriatal connectivity. We found that decreased dopamine activity was associated with functional alterations, mainly between the caudate seed and areas of the dopaminergic cortex, such as the frontal lobe, limbic system, and cerebellum (Figure 3). For example, dopamine depletion disrupts basal ganglia outflow and consequently affects the expression of prefrontal executive functioning by interrupting frontostriatal circuitry in PD (31). Our results were further strengthened by showing correlations between decreased numbers of striatofrontal network connections and increased disease severity and duration (Figure 4). Our results are open to several interpretations. Optimal dopamine levels are important, especially for neuroplasticity in the prefrontal cortices and the limbic system (32). Dopamine neuron degradation thus directly or indirectly disrupts both adjacent and distant areas of connection that share vulnerability to an altered neurotransmitter-related neuronal circuitry. In addition, sustained attenuation of dopamine activity weakens the protective effect of dopamine against glutamate-induced excitotoxicity. Abundant glutamate receptors within the frontal cortex, limbic system, and basal ganglia circuitry might account for cognitive and motor alterations in patients with PD (33).

Consistent with a previous structural covariance study (23), this functional network study revealed that the number of connections between the Vsi of the caudate and limbic system (including the cingulate gyrus, parahippocampus, insula, and temporal lobe) are increased in patients with PD. A recent PD longitudinal study also showed increased limbic system gray matter volume (34). Meanwhile, a report showed reduced dopamine transporters in the striatum and limbic system in PD patients with depression (35). Although our functional network results did not correlate with clinical presentation of the PD patients, this observation might also suggest compensation by the anterior striatum for the diseased posterior striatum.

We found increases in the striatocerebellar connectivity in some of the patients with PD while also finding decreases in such connectivity in others. Some of our findings were consistent with those of previous functional network and structural covariance studies (23, 36). In fact, both pathological and compensatory effects of the cerebellum have been observed in PD (10). Dopaminergic degeneration with subsequent structural damage and functional disconnection can account for the decrease in connectivity (10). Functional MRI studies showed weakened striatal–cortical and striatal–cerebellar connections causing basal ganglia dysfunction. The weakening of these connections can result, however, in compensatory strengthening of connections between corticocerebellar motor regions (36). In the present study, our finding of an association between decreased striatal dopamine levels and increased Vsi–cerebellar functional connectivity is consistent with the finding of cerebellar hyperactivity in PD patients by a previous motor task study (37). However, whether cerebellar hyperactivity is pathological or compensatory and how it affects phenotypes in PD patients remain open questions. We believe that a balance between different neurotransmitter alterations in PD might contribute to function modulation. Yet, this concept requires testing in further studies.

### Limitations

The results presented here should be interpreted with caution. It is possible, for example, that the observed group differences reflect long-term therapeutic effects rather than the effects of PD itself. Furthermore, the lack of any SPECT data for the NC group was another limitation of this study, one that is related to the reluctance of NCs to undergo SPECT scans. In any case, the lack of a direct comparison in this regard of the treated group to the untreated group may be considered a limitation. Some factors affecting image quality may have partially affected the study evaluation. Motion-related artifacts can affect rs-fMRI studies of PD patients in OFF medication status, and while the effects can be reduced using regression techniques, the effectiveness of this strategy is uncertain (38). Otherwise, our study failed to explore the relationships among dopamine neuron loss, putamen-associated networks, and clinical presentation. Since the HY stage or SE-ADL test was designed to evaluate general disease severity rather than specific functional domains in PD, our correlation results might reflect an evaluation bias for the frontal lobe, cerebellum, and other regions with functional network differences between the groups. In addition, the longer disease duration (mean 4.03 ± 4.20 years) in the patients with affected extensive extra-striatal GM makes it difficult to evaluate early putamen-associated motor network alterations in the present study. The study nonetheless partially reveals the natural course of PD under medical control and may be reinforced by further investigations using detailed clinical and neuropsychological examinations.

## Conclusion

We report a significant association between decreased striatal dopamine transporter uptake and changes in particular striatocortical networks, indicating that the functional neuroplasticity of different striatal subregions parallels the progression of striatal dopaminergic dysfunction in PD. The decrease in frontostriatal fc-cc also correlates with disease duration and clinical disease severity, suggesting a need for further evaluation of fc-cc as an objective marker for disease progression in future studies of putative disease-modifying therapies.

## Ethics Statement

The study was approved by the Institutional Review Board of Chang Gung Memorial Hospital in Taiwan.

## Author Contributions

W-CL: acquisition of data, study concept and design, and acquisition of data. T-WH: analysis and interpretation of data. C-CH: analysis and interpretation of data. Y-CH: acquisition of data. N-WT: acquisition of data. H-LC: analysis and interpretation of data. C-HL: acquisition of data, study concept, and critical revision of manuscript for intellectual content.

## Conflict of Interest Statement

The authors declare they have and have not had any actual or potential conflicts of interest, including any financial, personal, or other relationships, with other people or organizations within 3 years of beginning the submitted work that could inappropriately influence, or be perceived to influence, the work.

## Supplementary Material

Figure S1 Comparison of the functional connectivity maps of the caudate (overlap from the VSs, VSi, and dorsal caudate) and putamen (overlap from the dorsal caudal putamen, dorsal rostral putamen, and ventral rostral putamen) between Parkinson’s disease (PD) and normal control participants. Maps showing significant functional connectivity differences between groups were rendered on a structural T1 magnetic resonance template. The statistical threshold was achieved with a voxel-wise *P*_corrected_ < 0.05 based on Monte Carlo simulations. The corrected threshold corresponds to *P*_uncorrected_ < 0.01 with a minimum cluster size of 40 voxels for multiple comparisons.
