# Supplementary material for: Correlation between Dopamine Transporter Degradation and Striatocortical Network Alteration in Parkinson’s Disease
Source: Front Neurol. 2017 Jul 17;8:323. doi: 10.3389/fneur.2017.00323 (PMC5511968; doi:10.3389/fneur.2017.00323)
Supplement: Supplementary file 1 [file data_sheet_1.docx]

**2.3 Tc-99m TRODAT-1 Brain SPECT/CT data acquisition and preprocessing**

Data acquisition

The examination of Tc-99m TRODAT-1 Brain SPECT/CT, post-processing, and ROI evaluation in patients with PD were as previously reported([Hsu et al., 2014](#_ENREF_15)). Each patient with PD was intravenously injected with a 925-MBq (25 mCi) dose of Tc-99m TRODAT-1 (Institute of Nuclear Energy Research, Lung-Tan, Taiwan). The brain SPECT and CT scans were done 4 h later consecutively, using a hybrid SPECT/CT system (Symbia T; Siemens Medical Solutions, Hoffman Estates, IL, USA) with patients lying stably in a supine position with the head resting in a holder. The SPECT/CT system integrates a dual head SPECT camera with a two-slice spiral CT installed within the same gantry. The SPECT images were acquired in 120 steps (30 s per step) over a circular 360° rotation using low-energy, high-resolution parallel-hole collimators, a 128 × 128 matrix, and a ×1.45 zoom. The CT images were acquired without contrast medium using the following parameters: 130 kV; 45 mAs (Image Quality Reference mAs, CARE Dose 4D; Siemens Medical Solutions); rotation time, 1.5 s; collimation, 2 × 2.5 mm.

Image processing and ROI delineation

CT images were reconstructed to a 512 × 512 image matrix with a very small smoothing kernel, H08s (Siemens Medical Solutions) for SPECT attenuation correction, and a larger smoothing kernel, H30s (Siemens Medical Solutions), for image fusion and ROI delineation. Raw SPECT data were reconstructed into transaxial slices using flash 3D (OSEM reconstruction method with 3D collimator beam modeling) with 8 subsets, 8 iterations, and correction with the H08s CT attenuation map. Images were smoothed using a 3D spatial Gaussian filter (full width at half maximum, 6 mm).The reconstructed transaxial slice thickness was 3.3 mm. Reorientation with sagittal slices parallel to the anterior commissure-posterior commissure (AC-PC) line and correction for transaxial and coronal slice deviation was done manually by inspecting the SPECT images. Three consecutive SPECT transaxial slices showing the highest striatal uptake were summed. For manual SPECT ROI delineation, ROIs of the striatum, caudate, putamen, and occipital cortex were manually delineated directly on the summed SPECT image. The principal investigator reviewed all of the SPECT scans to determinate the ROI delineation. In equivocal cases, a second observer made the review. Both were blinded to the laboratory results at the time of clinical and imaging assessment.

The SPECT and CT images were coregistered using an autoregistration model on the manufacturer’s nuclear medicine workstation (SyngoMI workplace, version VA60B; Siemens Healthcare). Reorientation was also done manually by inspecting the fused SPECT/CT images with identical parameters (𝑥, 𝑦, 𝑧) for SPECT and CT images. Three consecutive SPECT transaxial slices showing the highest striatal uptake were summed. One CT slice with the most recognizable striatum was chosen for ROI drawing. CT-guided ROIs of the striatum, caudate, putamen, and occipital cortex were manually drawn on the CT images by observers who were blinded to the SPECT or fused SPECT/CT images. These CT-guided ROIs were then transferred to the coregistered summed SPECT image.

Striatal and subregional TRODAT-1 BP_ND_

We used the low DAT concentration area of the occipital cortex as a background ROI. The striatal, caudate, and putamen nondisplaceable binding potential (BP_ND_) were calculated by subtracting the mean counts of the occipital cortex ROI from the mean counts of the striatal, caudate, and putamen ROIs and dividing the result by the mean counts of the occipital cortex ROI([Hsu et al., 2014](#_ENREF_15)).

**2.4 MR data acquisition and preprocessing**

Resting-state functional MRI preprocessing and individual analyses

Prior to pre-processing, the first 10 volumes were discarded to reach a steady-state magnetization and allow the participants to adapt to the scanning noise. Resting-state fMRI data preprocessing was then performed using the Statistical Parametric Mapping software package (SPM8, Wellcome Department of Cognitive Neurology, London, UK; http://www.fil.ion.ucl.ac.uk/spm/) and Data Processing Assistant for Resting-State fMRI (DPARSF) tools ([Chao-Gan and Yu-Feng, 2010](#_ENREF_5)). Based on the recorded motion correction estimates, the subjects with more than 2 mm maximum displacement in any of the x, y, or z directions or more than 2° of angular rotation about any axis for any of the 190 volumes were excluded from this study. Data were also visually inspected for movement-related artifacts. The standard Montreal Neurological Institute template provided by SPM was further used for normalization with re-sampling to 3 mm cubic voxels and a Gaussian kernel of 6 mm (full-width at half maximum) for spatial smoothing. The waveform of each voxel was finally used to remove the linear trend of time course and for temporal band-pass filtering (0.01 to 0.08 Hz) to reduce low-frequency drift and high-frequency physiological noise ([Biswal et al., 1995](#_ENREF_2)). Physiological and other non-neuronal artifacts were reduced by regressing out the effects of predictors of the rs-fMRI data signal. Ten predictors were identified including respiration, cardiac, white matter (WM), cerebral spinal fluid (CSF), and 6 motion parameters. The physiological nuisance covariates, respiration and cardiac artifacts, were removed by means of PESTICA (Physiologic EStimation by Temporal ICA; http:// [www.nitrc.org/projects/pestica/](http://www.nitrc.org/projects/pestica/)). This is a data-driven estimator of cardiac and respiratory effects that uses the Infomax algorithm with enforced temporal independence ([Beall, 2010](#_ENREF_1)). This approach identifies cardiac and respiratory time series regressors, based on spatial weighting maps of cardiac and respiratory effects. For each subject's dataset, we manually estimated the spectral peak range nearest to the suggested maxima of 17 bpm and 60 bpm (respiratory and cardiac peaks, respectively).

The T1-weighted anatomical image was separated into WM and CSF maps using the SPM8 package (Wellcome Department of Cognitive Neurology). The resulting segmented WM and CSF masks were generated to ensure 80% probability of each tissue type. These individual tissue masks were then applied to the time series of each participant; the estimated predictors were calculated by averaging the time courses across all voxels within the mask.

**REFERENCES**

Beall, E.B. (2010). Adaptive cyclic physiologic noise modeling and correction in functional MRI. *Journal of neuroscience methods* 187(2)**,** 216-228.

Biswal, B., Yetkin, F.Z., Haughton, V.M., and Hyde, J.S. (1995). Functional connectivity in the motor cortex of resting human brain using echo-planar MRI. *Magn Reson Med* 34(4)**,** 537-541.

Butz, M., Worgotter, F., and van Ooyen, A. (2009). Activity-dependent structural plasticity. *Brain Res Rev* 60(2)**,** 287-305. doi: 10.1016/j.brainresrev.2008.12.023

S0165-0173(08)00151-3 [pii].

Cardoso, E.F., Fregni, F., Maia, F.M., Melo, L.M., Sato, J.R., Cruz, A.C., Jr., et al. (2010). Abnormal visual activation in Parkinson's disease patients. *Mov Disord* 25(11)**,** 1590-1596. doi: 10.1002/mds.23101.

Chao-Gan, Y., and Yu-Feng, Z. (2010). DPARSF: a MATLAB toolbox for ‚Äúpipeline‚Äù data analysis of resting-state fMRI. *Frontiers in Systems Neuroscience* 4.

Chaudhuri, K.R., Healy, D.G., Schapira, A.H., and National Institute for Clinical, E. (2006). Non-motor symptoms of Parkinson's disease: diagnosis and management. *Lancet Neurol* 5(3)**,** 235-245. doi: 10.1016/S1474-4422(06)70373-8.

Chou, K.H., Lin, W.C., Lee, P.L., Tsai, N.W., Huang, Y.C., Chen, H.L., et al. (2015). Structural covariance networks of striatum subdivision in patients with Parkinson's disease. *Hum Brain Mapp* 36(4)**,** 1567-1584. doi: 10.1002/hbm.22724.

Di Martino, A., Scheres, A., Margulies, D.S., Kelly, A.M., Uddin, L.Q., Shehzad, Z., et al. (2008). Functional connectivity of human striatum: a resting state FMRI study. *Cereb Cortex* 18(12)**,** 2735-2747. doi: bhn041 [pii]

10.1093/cercor/bhn041.

Draganski, B., Kherif, F., Kloppel, S., Cook, P.A., Alexander, D.C., Parker, G.J., et al. (2008). Evidence for segregated and integrative connectivity patterns in the human Basal Ganglia. *J Neurosci* 28(28)**,** 7143-7152. doi: 10.1523/JNEUROSCI.1486-08.2008

28/28/7143 [pii].

Folstein, M.F., Folstein, S.E., and McHugh, P.R. (1975). "Mini-mental state". A practical method for grading the cognitive state of patients for the clinician. *J Psychiatr Res* 12(3)**,** 189-198. doi: 0022-3956(75)90026-6 [pii].

Fox, M.D., and Greicius, M. (2010). Clinical applications of resting state functional connectivity. *Front Syst Neurosci* 4**,** 19. doi: 10.3389/fnsys.2010.00019.

Gasser, T., Bressman, S., Durr, A., Higgins, J., Klockgether, T., and Myers, R.H. (2003). State of the art review: molecular diagnosis of inherited movement disorders. Movement Disorders Society task force on molecular diagnosis. *Mov Disord* 18(1)**,** 3-18. doi: 10.1002/mds.10338.

Goetz, C.G., Poewe, W., Rascol, O., Sampaio, C., Stebbins, G.T., Counsell, C., et al. (2004). Movement Disorder Society Task Force report on the Hoehn and Yahr staging scale: status and recommendations. *Mov Disord* 19(9)**,** 1020-1028. doi: 10.1002/mds.20213.

Helmich, R.C., Derikx, L.C., Bakker, M., Scheeringa, R., Bloem, B.R., and Toni, I. (2010). Spatial Remapping of Cortico-striatal Connectivity in Parkinson's Disease. *Cerebral Cortex* 20(5)**,** 1175-1186. doi: DOI 10.1093/cercor/bhp178.

Hsu, C.C., Chang, Y.H., Lin, W.C., Tang, S.W., Wang, P.W., Huang, Y.C., et al. (2014). The feasibility of using CT-guided ROI for semiquantifying striatal dopamine transporter availability in a hybrid SPECT/CT system. *ScientificWorldJournal* 2014**,** 879497. doi: 10.1155/2014/879497.

Hughes, A.J., Daniel, S.E., Kilford, L., and Lees, A.J. (1992). Accuracy of clinical diagnosis of idiopathic Parkinson's disease: a clinico-pathological study of 100 cases. *J Neurol Neurosurg Psychiatry* 55(3)**,** 181-184.

Hwang, W.J., Yao, W.J., Wey, S.P., and Ting, G. (2004). Reproducibility of 99mTc-TRODAT-1 SPECT measurement of dopamine transporters in Parkinson's disease. *J Nucl Med* 45(2)**,** 207-213.

Jia, X., Liang, P., Li, Y., Shi, L., Wang, D., and Li, K. (2015). Longitudinal Study of Gray Matter Changes in Parkinson Disease. *AJNR Am J Neuroradiol* 36(12)**,** 2219-2226. doi: 10.3174/ajnr.A4447.

Leh, S.E., Petrides, M., and Strafella, A.P. (2010). The neural circuitry of executive functions in healthy subjects and Parkinson's disease. *Neuropsychopharmacology* 35(1)**,** 70-85. doi: 10.1038/npp.2009.88.

Lin, K.N., Wang, P.N., Liu, C.Y., Chen, W.T., Lee, Y.C., and Liu, H.C. (2002). Cutoff scores of the cognitive abilities screening instrument, Chinese version in screening of dementia. *Dement Geriatr Cogn Disord* 14(4)**,** 176-182. doi: dem14176 [pii].

Martinez-Martin, P., Rodriguez-Blazquez, C., Kurtis, M.M., Chaudhuri, K.R., and Group, N.V. (2011). The impact of non-motor symptoms on health-related quality of life of patients with Parkinson's disease. *Mov Disord* 26(3)**,** 399-406. doi: 10.1002/mds.23462.

Middleton, F.A., and Strick, P.L. (2000). Basal ganglia output and cognition: evidence from anatomical, behavioral, and clinical studies. *Brain Cogn* 42(2)**,** 183-200. doi: 10.1006/brcg.1999.1099

S0278-2626(99)91099-0 [pii].

Monchi, O., Petrides, M., Mejia-Constain, B., and Strafella, A.P. (2007). Cortical activity in Parkinson's disease during executive processing depends on striatal involvement. *Brain* 130(Pt 1)**,** 233-244. doi: 10.1093/brain/awl326.

Neufeld, J., Teuchert-Noodt, G., Grafen, K., Winter, Y., and Witte, A.V. (2009). Synapse plasticity in motor, sensory, and limbo-prefrontal cortex areas as measured by degrading axon terminals in an environment model of gerbils (Meriones unguiculatus). *Neural Plast* 2009**,** 281561. doi: 10.1155/2009/281561.

Owen, A.M. (2004). Cognitive dysfunction in Parkinson's disease: the role of frontostriatal circuitry. *Neuroscientist* 10(6)**,** 525-537. doi: 10.1177/1073858404266776.

Park, S.Q., Kahnt, T., Talmi, D., Rieskamp, J., Dolan, R.J., and Heekeren, H.R. (2012). Adaptive coding of reward prediction errors is gated by striatal coupling. *Proc Natl Acad Sci U S A* 109(11)**,** 4285-4289. doi: 10.1073/pnas.1119969109

1119969109 [pii].

Politis, M. (2014). Neuroimaging in Parkinson disease: from research setting to clinical practice. *Nat Rev Neurol* 10(12)**,** 708-722. doi: 10.1038/nrneurol.2014.205.

Poston, K.L., and Eidelberg, D. (2012). Functional brain networks and abnormal connectivity in the movement disorders. *Neuroimage* 62(4)**,** 2261-2270. doi: 10.1016/j.neuroimage.2011.12.021

S1053-8119(11)01423-6 [pii].

Postuma, R.B., and Dagher, A. (2006). Basal ganglia functional connectivity based on a meta-analysis of 126 positron emission tomography and functional magnetic resonance imaging publications. *Cereb Cortex* 16(10)**,** 1508-1521. doi: bhj088 [pii]

10.1093/cercor/bhj088.

Power, J.D., Barnes, K.A., Snyder, A.Z., Schlaggar, B.L., and Petersen, S.E. (2012). Spurious but systematic correlations in functional connectivity MRI networks arise from subject motion. *Neuroimage* 59(3)**,** 2142-2154. doi: 10.1016/j.neuroimage.2011.10.018.

Remy, P., Doder, M., Lees, A., Turjanski, N., and Brooks, D. (2005). Depression in Parkinson's disease: loss of dopamine and noradrenaline innervation in the limbic system. *Brain* 128(Pt 6)**,** 1314-1322. doi: 10.1093/brain/awh445.

Schwab RS, and Engeland A (1969). *Projection technique for evaluating surgery in Parkinson's disease.* Edinburgh: E and S Livingstone.

Seeley, W.W., Crawford, R.K., Zhou, J., Miller, B.L., and Greicius, M.D. (2009). Neurodegenerative diseases target large-scale human brain networks. *Neuron* 62(1)**,** 42-52. doi: 10.1016/j.neuron.2009.03.024

S0896-6273(09)00249-9 [pii].

Tritsch, N.X., and Sabatini, B.L. (2012). Dopaminergic modulation of synaptic transmission in cortex and striatum. *Neuron* 76(1)**,** 33-50. doi: 10.1016/j.neuron.2012.09.023

S0896-6273(12)00858-6 [pii].

Vaarmann, A., Kovac, S., Holmstrom, K.M., Gandhi, S., and Abramov, A.Y. (2013). Dopamine protects neurons against glutamate-induced excitotoxicity. *Cell Death Dis* 4**,** e455. doi: 10.1038/cddis.2012.194

cddis2012194 [pii].

Ward, B.D. (2000). Simultaneous inference for fMRI data. *AFNI 3dDeconvolve Documentation, Medical College of Wisconsin*.

Wu, T., and Hallett, M. (2013). The cerebellum in Parkinson's disease. *Brain* 136(Pt 3)**,** 696-709. doi: 10.1093/brain/aws360

aws360 [pii].

Wu, T., Wang, L., Hallett, M., Chen, Y., Li, K., and Chan, P. (2011). Effective connectivity of brain networks during self-initiated movement in Parkinson's disease. *Neuroimage* 55(1)**,** 204-215. doi: 10.1016/j.neuroimage.2010.11.074

S1053-8119(10)01552-1 [pii].

Yu, H., Sternad, D., Corcos, D.M., and Vaillancourt, D.E. (2007). Role of hyperactive cerebellum and motor cortex in Parkinson's disease. *Neuroimage* 35(1)**,** 222-233. doi: 10.1016/j.neuroimage.2006.11.047.
