# Supplementary material for: Correlation between Dopamine Transporter Degradation and Striatocortical Network Alteration in Parkinson’s Disease
Source: Front Neurol. 2017 Jul 17;8:323. doi: 10.3389/fneur.2017.00323 (PMC5511968; doi:10.3389/fneur.2017.00323)

Significant clusters in the Two-sample analysis comparing functional connectivity in Parkinson's diease patients.

| Seed | functional Maps | Cluster anatomical locations | Cluster size | Primary peak location | T-value |
| --- | --- | --- | --- | --- | --- |
| RT DC |  |  |  |  |  |
|  | **Notreatment >treament** | Hippocampus_L (aal) | 61 | (-30 -21 -18) | 3.9993 |
|  | **Notreatment < treament** | Supp_Motor_Area_L (aal) | 51 | (-9 9 63) | 4.2952 |
| LT DRP |  |  |  |  |  |
|  | **Notreatment >treament** | Temporal_Inf_R (aal) | 190 | (48 -60 -21) | 3.8724 |
|  |  | Temporal_Inf_L (aal) | 307 | (-36 -57 -6) | 4.6855 |


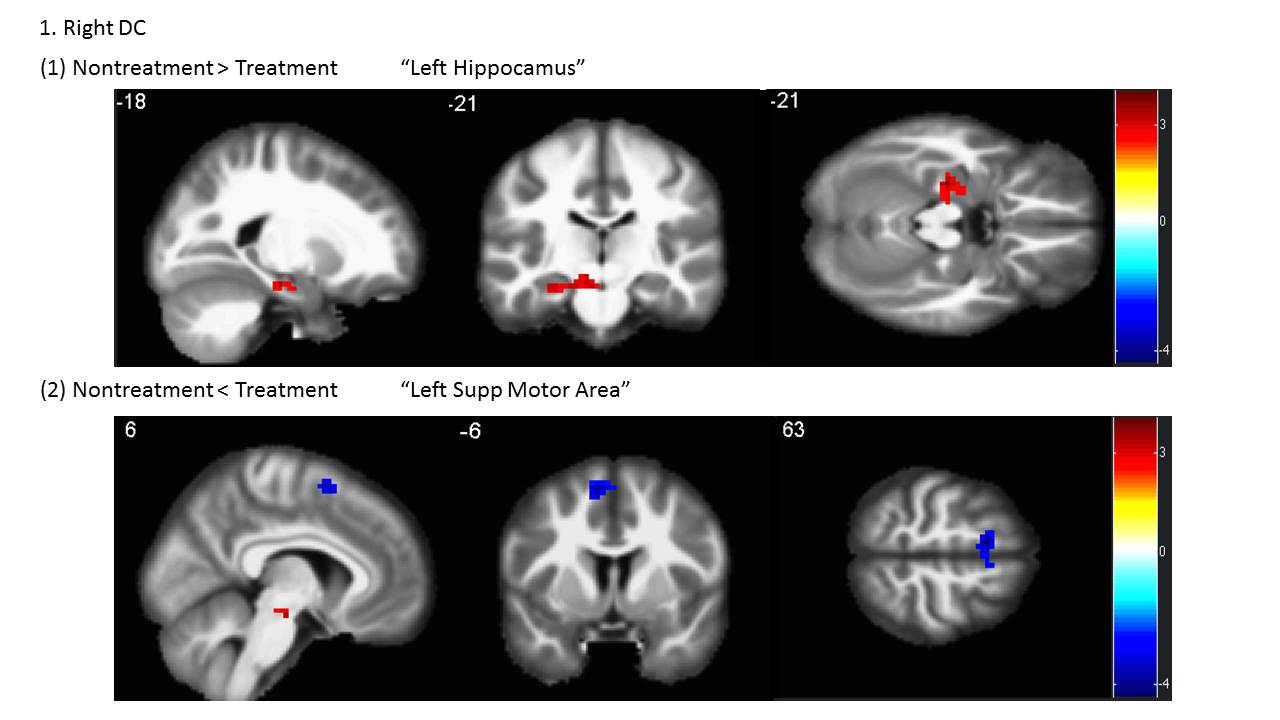


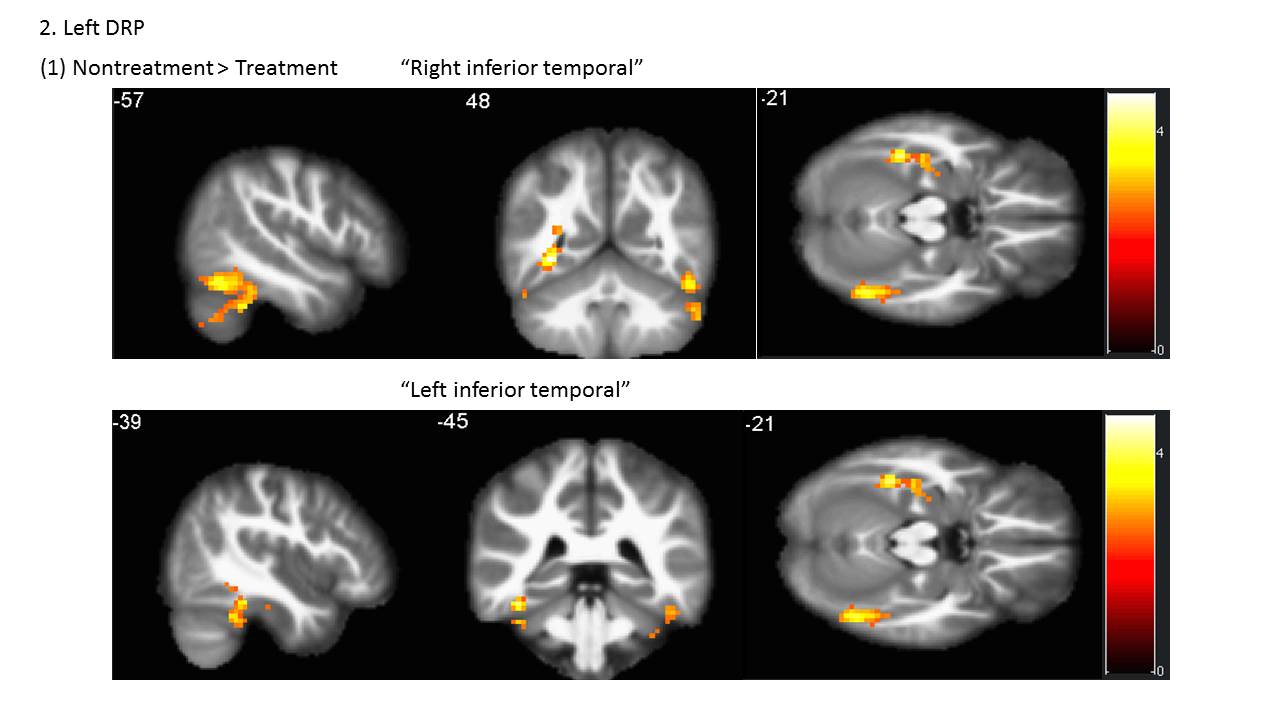

Supplement: Supplementary file 2 [file data_sheet_2.docx]
