# Supplementary figures and images for: Correlation between Dopamine Transporter Degradation and Striatocortical Network Alteration in Parkinson’s Disease
Source: Front Neurol. 2017 Jul 17;8:323. doi: 10.3389/fneur.2017.00323 (PMC5511968; doi:10.3389/fneur.2017.00323)

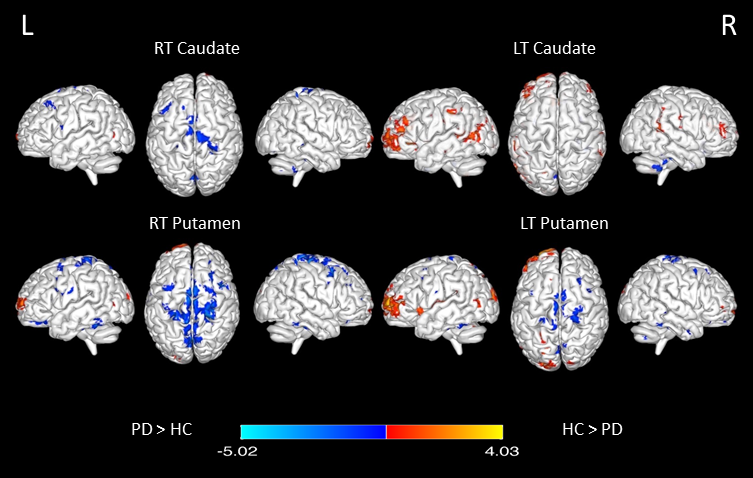

Supplement: Figure S1 — Comparison of the functional connectivity maps of the caudate (overlap from the VSs, VSi, and dorsal caudate) and putamen (overlap from the dorsal caudal putamen, dorsal rostral putamen, and ventral rostral putamen) between Parkinson’s disease (PD) and normal control participants. Maps showing significant functional connectivity differences between groups were rendered on a structural T1 magnetic resonance template. The statistical threshold was achieved with a voxel-wise Pcorrected < 0.05 based on Monte Carlo simulations. The corrected threshold corresponds to Puncorrected < 0.01 with a minimum cluster size of 40 voxels for multiple comparisons. [file image_1.tif]
